# Enhancing Shelf Life Prediction of Fresh Pizza with Regression Models and Low Cost Sensors

**DOI:** 10.3390/foods12061347

**Published:** 2023-03-22

**Authors:** Paul Wunderlich, Daniel Pauli, Michael Neumaier, Stephanie Wisser, Hans-Jürgen Danneel, Volker Lohweg, Helene Dörksen

**Affiliations:** 1 inIT–Institute Industrial IT, OWL University of Applied Sciences and Arts, 32657 Lemgo, Germany; 2 Institute for Life Science Technologies (ILT.NRW), OWL University of Applied Sciences and Arts, 32657 Lemgo, Germany

**Keywords:** spoilage prediction, regression models, low-cost sensors, machine learning, sustainability, food waste

## Abstract

The waste of food presents a challenge for achieving a sustainable world. In Germany alone, over 10 million tonnes of food are discarded annually, with a worldwide total exceeding 1.3 billion tonnes. A significant contributor to this issue are consumers throwing away still edible food due to the expiration of its best-before date. Best-before dates currently include large safety margins, but more precise and cost effective prediction techniques are required. To address this challenge, research was conducted on low-cost sensors and machine learning techniques were developed to predict the spoilage of fresh pizza. The findings indicate that combining a gas sensor, such as volatile organic compounds or carbon dioxide, with a random forest or extreme gradient boosting regressor can accurately predict the day of spoilage. This provides a more accurate and cost-efficient alternative to current best-before date determination methods, reducing food waste, saving resources, and improving food safety by reducing the risk of consumers consuming spoiled food.

## 1. Introduction

A major concern of the global Sustainable Development Goals (SDGs) is to counteract food wastage (Goal 12.3) [1]. In Germany alone, about 10 million tonnes of edible food are thrown away every year for a variety of reasons [2]. Worldwide, about 1.3 billion tonnes of food are lost or wasted [3]. A decisive share of 59% (6.5 million tonnes) is generated in private households. There in turn, 30% of food is thrown away due to an expired best-before date (BBD) [4].

The best-before date assures the end consumer that the food will retain its specific properties (e.g., taste, colour and consistency) under appropriate storage conditions and can still be consumed without hesitation, taking into account all influences along the food supply chain [5]. Based on various storage tests and quality controls, manufacturers establish a time safety buffer, which is intended to mitigate potentially less than ideal handling during transport or out-of-production storage. This gives the food producer in and outside Europe the certainty that the product still has the promised specific properties at the end of the best-before date [6]. However, due to the safety buffer, these food products can often still be consumed after the expiration of the best-before date. The technical processes in food processing are dependent on the individual raw material quality and on fluctuations in the course of processing. Therefore, depending on the batch, there are also individual risks of chemical or microbial spoilage, allergen or contaminant risks [7].

In an era of diminishing resource availability and in light of the European Union targets for sustainable food systems [8], there is a need for innovative but also economically viable solutions to improve best-before date prediction. The application of machine learning can help here, to reduce overall food waste by improving predictions of the best before dates listed on food packaging. A valuable contribution to food safety is also made by the mechanisms that must be integrated for this purpose such as such as data collection for real-time quality control in production.

Therefore, we came up with the following research questions.

I. What are examples of the use of machine learning in the food industry regarding spoilage detection and what are their limitations?
II. What types of sensors provide the most relevant and accurate data for determining the shelf life of fresh pizza?
III. What machine learning algorithm can be effectively utilized to predict the shelf life of fresh pizza using sensor data?

The research aims to demonstrate the feasibility of incorporating low-cost sensors into food shelf-life prediction prognostics through a use case involving fresh pizza stored in a refrigerator. The complex food matrix of pizza provides a suitable case for evaluating the performance of low-cost sensors such as gas sensors, ethanol, pH and near-infrared spectroscopy. A prediction concept is developed based on the collected sensor data and machine learning models are applied to predict the shelf-life of the fresh pizza. The models are evaluated utilizing established machine learning metrics to determine their efficacy and accuracy.

## 2. Relevant Work

This chapter provides an overview of the state of spoilage in ready-to-eat pizza and the integration of machine learning in the food industry. The latest research findings and technological innovations are presented, with a focus on the use of machine learning for enhancing spoilage detection and prevention in pizza and other food products.

Most studies in the field of food technology in relation to ready-to-eat pizza are aimed at the course of the spoilage process and whether a food is still edible. Singh, Wani and Goyal [9,10,11] deal with the effects of different modified atmospheres during refrigerated storage on the sensory, microbiological and chemical properties, as well as quality and shelf life of a homemade vegetarian ready-to-bake pizza. A shelf life study and environmental monitoring of pizza base with tomato puree under modified atmospheres was investigated by Fasano and Gallo [12]. In this study, the pizza base with tomato puree was examined microbiologically and for gas concentration changes. Their spoilage processes were also investigated for other components of a ready-made pizza, such as mozzarella cheese. Alves et al. [13] conducted a study on the sensory properties of letter-cut mozzarella cheese under different modified atmospheres, including its odour, taste and overall quality. Alam and Goyal [14] investigated the effects of different packaging materials and modified atmospheres on the microbiological quality of home-made mozzarella cheese during refrigerated storage. Likewise, different types of vegetables have also been studied, such as courgettes or peppers. This work primarily looks at how certain packaging materials or packaging strategies affect shelf life. In particular, sensory and chemical properties as well as gas concentration were analysed. The examination of courgettes by Lucera et al. [15] also included additional analysis of microbiological contamination. The testing of the green bell peppers by Manolopoulou et al. [16] included the measurement of colour indices. Oliveira et al. [17] dealt with the influence of temperature and the number of foil perforations for sliced mushrooms. They evaluated the quality of the mushrooms and developed a kinetic shelf-life model for modified atmosphere packaging. Investigations into the spoilage process of a ready-to-eat pizza and its components are currently mostly limited to basic investigations in the laboratory. However, these laboratory analyses are time-consuming and very cost-intensive. Therefore, use of machine learning (ML) in the food industry has gained increasing popularity in recent years, as it offers a non-destructive and efficient way to evaluate the quality and safety of food products. ML techniques have been applied to various aspects of food evaluation, including classification of food products based on colour, texture, and chemical properties, as well as the detection of defects and contamination. For instance, Ireri et al. [18] employed support vector machines (SVM) to classify tomatoes based on their colour and texture features, with a focus on detecting defects and stains on their surface. Kanade et al. [19] used the K-Nearest Neighbour (KNN) algorithm to classify guava fruits. Liang et al. [20] proposed a separate fruit tray system and a deep learning-based method to detect and classify high-quality apples in real-time. Basak et al. [21] developed a non-destructive method using ML algorithms to predict the total soluble solids (TSS) and pH of strawberries. Image processing techniques have also been used in several studies to assess food quality. For example, Kumar et al. [22] utilized ML systems to evaluate the quality of pomegranate fruits, while Ropelewska et al. [23] distinguished fresh and lacto-fermented red bell pepper samples using image texture analysis and the KNN algorithm. Other researchers have employed more advanced technologies such as neural networks (NN). Basile et al. [24] used non-destructive NIR spectroscopy and ML to predict texture parameters and TSS content in intact berries. Xiong et al. [25] proposed a transfer-learning-based model using a 3D-printed electronic nose and deep learning to detect the freshness of chicken breasts. Kim et al. [26] proposed a deep learning-based Haugh unit (HU) prediction model to determine egg freshness using non-destructive weight loss measurements. The model uses a stacked convolutional neural network (CNN) and long short-term memory (LSTM) algorithm with data augmentation to improve the accuracy of HU prediction compared to traditional ML methods. Furthermore, some researchers have explored the use of ML combined with other technologies to evaluate food quality. For instance, Darwish et al. [27] proposed a novel approach combining microwave (MW) sensing technology and ML tools such as MLP and SVM to classify food products as contaminated or uncontaminated with high accuracy. Fengou et al. [28] investigated the use of FTIR spectroscopy and multispectral imaging in combination with ML algorithms to evaluate the microbiological quality of chicken burgers. Cheng et al. [29] utilized near-infrared spectroscopy and hyperspectral imaging data in a partial least squares regression to predict the chemical properties of fish muscle tissue. Faqeerzada et al. [30] investigated the use of shortwave-infrared hyperspectral imaging (SWIR-HSI) combined with the one-class classifier DD-SIMCA for high-throughput quality screening of almond powder regarding potential adulteration. Finally, Kang et al. [31] provide a comprehensive review of the current applications of machine learning and hyperspectral imaging in the food supply chain for non-destructive testing and evaluation of food quality and safety attributes. Özdoğan et al. [32] give a detailed overview of recent developments in hyperspectral imaging systems for determining sensory properties such as colour, defects, texture, taste, freshness, and ripeness in various foods. The authors note that the visible and near-infrared region is the most commonly used spectral range for sensory evaluation, and linear regression models are the most commonly used multivariate analysis techniques.

In conclusion, machine learning has shown to be a useful tool in the food industry for quickly and effectively assessing the quality and safety of food. Different sensors, including hyperspectral imaging, spectroscopy, and microwave sensing, have been combined with different machine learning techniques, such as support vector machines, K-nearest neighbors, deep learning, and neural networks, to detect flaws, classify food products, and predict chemical and sensory properties. These methods have been used on a variety of food items, including fruits, vegetables, meat, and dairy, with encouraging results in terms of spotting contaminants and determining ripeness.

## 3. Materials and Methods

**Figure 1:**
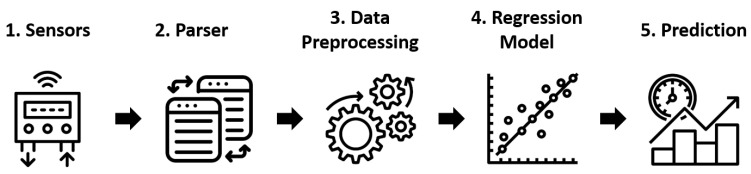
General Concept.

In order to make more accurate predictions regarding the shelf-life of fresh pizzas, several prerequisites must be met. To begin, data must be gathered for a prediction model that utilizes machine learning techniques. This data should encompass various characteristics of the fresh pizza, specifically those that change during the spoilage process. These characteristics can be measured using various sensor technologies and serve as the foundation for the data. A comprehensive overview of the overall concept is depicted in Figure 1.

The methodology for predicting the shelf-life of fresh pizzas involves five steps, starting from sensor data acquisition and culminating in the development and deployment of a machine learning model and its predictions. Once data are collected by the sensors, a parser is required to convert the sensor data from the manufacturer-specific file format to an uniform file format. After that, the data must be cleaned, transformed, and prepared for modeling. These stages will be discussed in greater detail subsequently. A regression model can then be trained to make predictions. Finally, these predictions must be evaluated and the regression model may need to be fine-tuned, as required.

### 3.1. Measurement Setup

**Figure 2:**
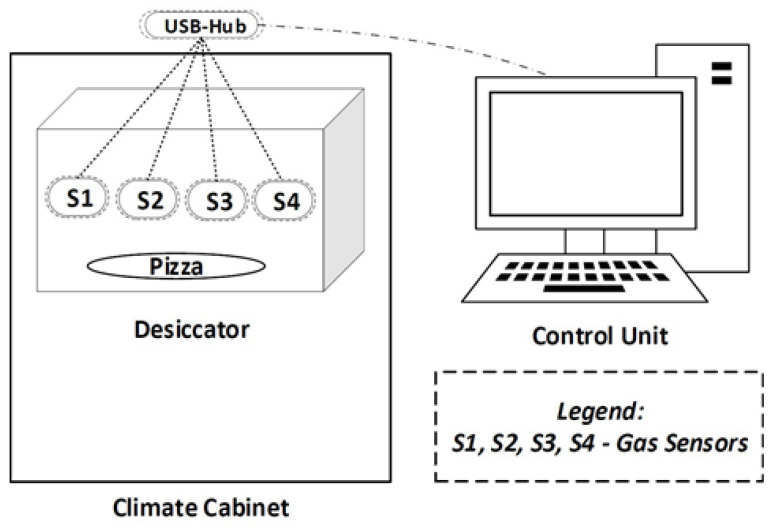
Measurement Setup.

The first step in the process is to acquire sensory information from the pizza using a specially designed measurement setup, as illustrated in Figure 2.

The measurement setup consists of the climate-controlled cabinet Binder MKF 115 E 3.1 [33] that houses a desiccator. The cabinet is specifically configured to maintain a temperature, which is also the temperature inside the desiccator. The desiccator is a nearly airtight container that provides a secure base for attaching sensors. In addition, the desiccator has the advantage of keeping the moisture well and protecting the product from drying out like a package. This allows sensor data to be collected over a longer period of time before the pizza dries out. After the appropriate storage period, the pizza slice is removed and the desiccator is carefully cleaned. The necessary sensors (S1, S2, S3 and S4) are strategically placed within the measurement setup and are remotely operated by a control unit. Data transfer between the sensors and Windows-based control unit is achieved via a USB hub and a USB cable.

### 3.2. Sensors

For the storage tests, the following sensors were used in the measurement setup:

- CO_2_ sensor
- VOC sensor
- Ethanol sensor
- pH sensor
- NIR sensor

#### 3.2.1. CO_2_ and VOC Sensors

The SCD30 sensor [34] is a carbon dioxide (CO_2_) sensor and the SVM40 sensor [35] is a volatile organic compound (VOC) sensor. Both sensors are developed by the manufacturer Sensirion, located in Stäfa, Switzerland. They are controlled via the “SEK-ControlCenter” software in our storage test. The SCD30 sensor is a non-dispersive infrared sensor (NDIR sensor) with a measurement range of 400 ppm–10,000 ppm. It takes measurements every two seconds and is connected to a sensor bridge via Ethernet cable, which is then connected to the control unit. The SVM40 sensor is built on the concept of a metal oxide semiconductor sensor and is connected to a computer via UART (Universal Asynchronous Receiver-Transmitter) and USB-C connection. The sensor is capable of measuring both the processed value and a digital raw value signal (SRAW VOC). A temperature and humidity sensor is included on both sensors.

#### 3.2.2. Ethanol Sensor

The GDX-ETHO sensor from Vernier [36] also relies on the principle of a metal oxide semiconductor sensor and can measure the ethanol content in the vapor phase. The sensor can be connected via Bluetooth 4.2 or a USB-C connection. Depending on the configuration using Vernier’s “Graphical Analysis^®^” software, the sensor measures in a predefined time interval in the unit ppm.

#### 3.2.3. pH Sensor

The MultiLine^®^ Multi 3620 IDS [37] pocket multi-parameter meter from Xylem Inc. (Washington, DC, USA) was used in combination with the SenTix^®^ Sp-T 900 [38] pH probe electrode to measure the pH value. This combination allows for measurement in a pH range of 2 to 13 with an accuracy of ±0.004. The measurement interval and period can be defined in advance. The pH electrode, designed for penetration measurement of semi-solid foods and placed at the edge of the dough, was re-calibrated with the appropriate buffer solution before each measurement.

#### 3.2.4. NIR Sensor

For optical measurement, a near-infrared sensor, the Tellspec Enterprise Sensor [39], was used. This sensor measures in a range of 900 to 1700 nanometers and is based on the NIR-S-G1 Sensor module from Inno Spectra [40]. It is powered by USB and controlled via the Inno Spectra Corporation NIR Scan software. The individual measurements result from an average of 50 individual measurements. The values recorded are absorption, reflection, and intensity.

### 3.3. Storage Tests

The storage tests were conducted using the measurement setup as described previously. In total, 12 storage tests were performed. Each storage test is a measurement series with one pizza sample. The climate chamber was set to a temperature of 5 °C and a relative humidity of 60%. The use case is a fresh pizza, which is richly topped with grilled courgette, grilled sweet pepper and mozzarella. The ingredients are wheat flour, 15% strained tomatoes, 12% grilled sweet peppers, 12% firm mozzarella cheese, water, 6.3% grilled courgettes, 3.1% grilled aubergines, 1.4% tomato concentrate, rapeseed oil, salt, baker’s yeast, extra virgin olive oil, sugar, oregano, parsley, garlic, onions, pepper, basil, fried onions. In its original state, it is a commercial frozen pizza suitable for vegetarians, which has been defrosted in the refrigerator before measurement. A representative sample of 40 g of a thawed and unopened pizza was placed in the desiccator, close to the necessary sensors. The sample was monitored and measured over a 14-day period under controlled temperature and humidity conditions. The desiccator’s temperature was approximately 7 °C and the relative humidity was approximately 70%. Measurements of NIR, pH and ethanol were taken every 20 min, while the CO_2_ sensor was set to measure every 2 s, and the VOC sensor was set to measure once per second.

### 3.4. Parsing and Data Preprocessing

The sensors have digitally captured the fresh pizza during the storage test. However, these data are typically in a manufacturer-specific format, which can make data processing more difficult. To ensure efficient and streamlined data processing, it is essential to have a uniform data format as a foundation. To achieve this, the csv file format was utilized. Parsers were developed and implemented to convert the sensor data into the csv file format for compatibility and ease of use. Next, steps of data preprocessing are carried out. Here, outliers and faulty measurements are eliminated and any missing information is imputed if necessary. The sensor data are not yet sufficient in this form to learn a regression model. Since regression is a supervised learning process, it requires not only features (sensor data), but also labels (target values) that serve as a target for learning. To achieve this, a label was created based on the shelf life of each pizza. The day of spoilage was defined by food experts and a minimum safety buffer of 1 day was additionally applied. The determination was carried out on pizzas from the same batch by means of microbiological and human sensory tests. For creating the labels, the day of spoilage is always set to day 0. All days up to the day of spoilage are positively decreasing. This means that a label of 7 means that there are still 7 days until the day of spoilage. For days after the day of spoilage, a negative decreasing count is applied: -1 for the first day after, -2 for the second day after, and so on. This means that the regression model is trained to determine the position on the time axis in relation to the day of spoilage.

### 3.5. Data

The data provided to the model consists of preprocessed values from various sensors, along with the aforementioned labels. In the following, we provide an overview of what each sensor was measuring. The CO_2_ sensor measures levels of carbon dioxide, temperature, and humidity. The VOC sensor measures levels of volatile organic compounds (VOCs), temperature. The ethanol sensor measures levels of ethanol in parts per million (ppm). The pH sensor measures pH value and temperature. Lastly, the NIR sensor measures absorption values for wavelengths between 900 and 1700 nm. As an example, we present a subset of the VOC data in Table 1.

The data from the other sensors follows a similar format.

## 4. Results

After completion of storage tests and preprocessing of the data, a machine learning model can now be trained to predict the day of spoilage using features and labels. Regression methods, being a type of supervised learning, are particularly well-suited for this task. In a supervised learning method, the model learns the relationship between the input variables (features) and output variable (label) by fitting a mathematical function to the data. The coefficients of the function are chosen to make the most accurate predictions of the label [41].

### 4.1. Regression Model Concept

**Figure 3:**
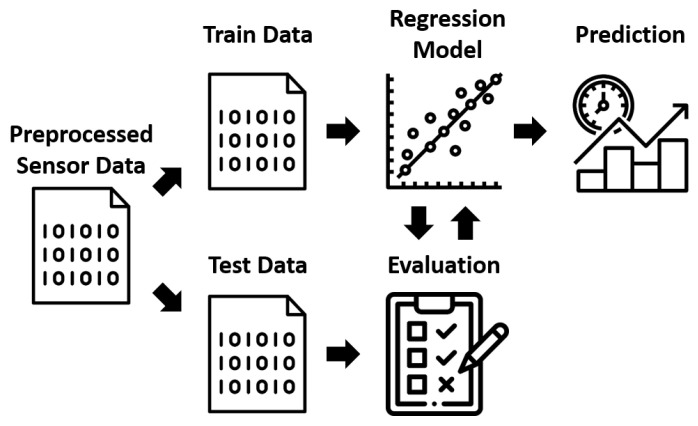
Regression Model Concept.

The concept for creating the regression model is illustrated in Figure 3.

Here, the preprocessed sensor data are divided into training data and test data. The training data are used as input for the regression method to create the regression model. The test data, in turn, serves as unknown and new data for the regression model. Using the test data, the model’s reliability on previously unknown data can be checked in an evaluation. Different metrics are calculated in the evaluation to determine the quality of the model. Based on these metrics, the regression model can be optimized by adjusting specific hyperparameters of the model. Once the regression model has been fully trained and optimized, it is ready for the application of prediction.

### 4.2. Regression Algorithms

Learning a regression model can be achieved through various algorithms. In this work, we compare and investigate the effectiveness of two specific methods, namely the Random Forest Regressor [42] and the XGBoost Regressor [43]. We evaluate their performance and suitability for the prediction of the day of spoilage. More complex machine learning methods, such as neural networks, were not explored because they require more time and computational power, and need significantly more labeled data for training. The labeling of data in the food industry is time-consuming and expensive. More practical and easier-to-understand methods, such as random forest and XGBoost, were preferred.

#### 4.2.1. Random Forest Regressor

The Random Forest Regressor [42] is a particular implementation of the Random Forest algorithm that is used for regression tasks. An ensemble learning technique called Random Forest makes predictions by using multiple decision trees. Each decision tree in a Random Forest Regressor ensemble is trained using a random subset of the training data, and the ensemble’s predictions are averaged to produce the final prediction. This approach facilitates in lowering overfitting and enhancing the model’s general accuracy.

The algorithm is presented in pseudo code in Algorithm 1, and it requires several inputs including the data set, the number of trees in the ensemble (n_estimators), the minimum number of samples required to split an internal node (min_samples_split), and the maximum depth of the trees (max_depth). These parameters are important for controlling the complexity and performance of the model. The first step of the algorithm is to extract the features (X) and the labels (Y) from the data set. Then, it splits the data into training and test sets with a ratio of 75 to 25. Then, the algorithm initializes an empty list “forest” to store decision trees and iteratively samples the training data with replacement. For each iteration, it fits a decision tree to the sample using min_samples_split as the number of samples required to split an internal node and appends the tree to the list. By using random subsets of the data and random subsets of features at each split, a random forest is able to reduce overfitting and improve generalization performance. After all decision trees are fitted, it creates a new random forest model, fit it using the list of decision trees. Finally, it returns the fitted model “rfr” as the output.
**Algorithm 1** Random Forest Regression.  1:**procedure** RandomForest(data,n_estimators,min_samples_split,max_depth)  2:    Extract features (X) and labels (Y) from the data  3:    $X_{train},X_{test},Y_{train},Y_{test}$← split(data, ratio = 0.75)  4:    Initialize an empty list forest to store decision trees  5:    **for** *i* in range n_estimators **do**  6:        Randomly sample $X_{train}^{\prime }$, $Y_{train}^{\prime }$ from $X_{train}$, $Y_{train}$ with replacement  7:        Fit a decision tree to $X_{train}^{\prime }$ and $Y_{train}^{\prime }$ with min_samples_split and max_depth  8:        Add the fitted decision tree to forest  9:    **end for**10:    Create a new random forest model rfr11:    Fit rfr using the decision trees in forest12:    **return** the fitted model rfr13:**end procedure**

A RandomizedsearchCV with cv = 5 was conducted for each Random Forest Regressor to optimize the hyperparameters n_estimators, max_depths and min_samples_split. The use of RandomizedsearchCV allows for an efficient search of the hyperparameter space, as it randomly samples a set of potential hyperparameters to evaluate. However, it should be noted that the search is not exhaustive, meaning there could be other combinations of hyperparameters that would yield better results. The optimized hyperparameters for each Random Forest Regressor are shown in Table 2.

#### 4.2.2. XGBoost Regressor

The XGBoost Regressor is a particular implementation of the XGBoost algorithm that is used for regression analysis. Regression is a supervised learning method that is used to predict a continuous outcome variable (also known as a response variable) based on one or more predictor variables. In the context of XGBoost, the regressor is trained using gradient boosting, which entails constructing a model by combining several weak learners (such as decision trees) and subsequently improving the model iteratively by modifying the weights of each learner based on the error in the preceding iteration. By using this approach, XGBoost is able to achieve highly accurate predictions on regression tasks. The XGBoost algorithm is outlined using pseudo code in Algorithm 2.
**Algorithm 2** XGBoost Regressor.1:**procedure** XGBoost(data,n_estimators,max_depth,learning_rate,gamma)2:    Extract features (X) and labels (Y) from the data3:    $X_{train},X_{test},Y_{train},Y_{test}$← split(data, ratio = 0.75)4:    Initialize the XGBoost model with the given parameters:5:    n_estimators, max_depth, learning_rate, gamma6:    Create a new XGBoost model xgbr7:    Fit xgbr on $X_{train},Y_{train}$8:    **return** the fitted model xgbr9:**end procedure**

The algorithm requires as input the sensor data (features and label) and the hyperparameters: the number of trees in the model (n_estimators), the maximum depth of the trees (max_depth), the learning rate (learning_rate) and the regularization term (gamma). For regulating the model’s complexity and effectiveness, these hyperparameters are crucial. The sensor data are divided into training and test sets with a ratio of 75 to 25. The algorithm then initializes the XGBoost model with the chosen hyperparameters and fits the model to the training set. Finally, the algorithm returns the fitted model “xgbr” as the output.

For optimizing the hyperparameters max_depth, learning_rate, n_estimators and gamma, a RandomizedsearchCV with cv = 5 was conducted for each XGBoost Regressor. The following optimized hyperparameters were obtained and are depicted in Table 3:

### 4.3. Evaluation

A Random Forest Regressor and an XGBoost Regressor were trained for each of the different sensors from Section 3.2. The models will be evaluated in the following, and their performance will be determined using various metrics. Listed below are the metrics that were utilized:

- R-Squared ($R^{2}$)
- Mean Squared Error (MSE)
- Root Mean Squared Error (RMSE)
- Mean Absolute Error (MAE)
- Symmetric Mean Absolute Percentage Error (SMAPE)

$R^{2}$ [44] is a statistical measure used to evaluate the goodness of fit of a regression. Its range of values goes from 0 to 1, where 1 represents a perfect model.

The Mean Squared Error (MSE) [45] is a metric commonly used in regression analysis. It measures the expected squared distance between the predicted values of the regression model and the true values. The formula for MSE is given by:

(1) $$\mathrm{MSE}=\frac{1}{n}\sum_{i=1}^{n}{\left(y_{i}-{\hat{y}}_{i}\right)}^{2}$$

where *n* is the number of observations, $y_{i}$ is the true value of the *i*-th observation, and ${\hat{y}}_{i}$ is the predicted value of the *i*-th observation. Although there is no absolute value to imply whether a model is good, MSE can be used to compare models to one another, as well as other metrics. The other metrics also hold true in this regard.

Root Mean Squared Error (RMSE) [46] is calculated by taking the square root of the mean squared errors. RMSE is a popular evaluation metric in regression analysis and machine learning. It is similar to MSE but easier to interpret since the RMSE value has the same scale as the predicted values. The formula for RMSE is given by:

(2) $$\mathrm{RMSE}=\sqrt{\frac{1}{n}\sum_{i=1}^{n}{\left(y_{i}-{\hat{y}}_{i}\right)}^{2}}$$

The Mean Absolute Error (MAE) [46] measures the average magnitude of the errors in a prediction, indicating how far off the predicted values are from the true values. It does not indicate the direction of the deviation. The formula for calculating MAE is given by:

(3) $$\mathrm{MAE}=\frac{1}{n}\sum_{i=1}^{n}\left|y_{i}-{\hat{y}}_{i}\right|$$

Here, *n* is the number of data points, $y_{i}$ is the true value for the *i*-th data point, and ${\hat{y}}_{i}$ is the predicted value for the *i*-th data point. By taking the absolute value of the difference between the true and predicted values and averaging over all data points, MAE provides a measure of the average magnitude of the errors in the predictions.

The Symmetric Mean Absolute Percentage Error (SMAPE) [47] is a measure of accuracy based on percentage errors, which can be calculated using the following equation:

(4) $$\mathrm{SMAPE}=\frac{1}{n}\sum_{i=1}^{n}\frac{2|y_{i}-{\hat{y}}_{i}|}{|y_{i}\left|+\right|{\hat{y}}_{i}|}\times 100\%$$

SMAPE is very similar to the Mean Absolute Percentage Error (MAPE) [48], but SMAPE is preferred in situations where estimates close to zero are important, as MAPE does not provide any information on such estimates. However, one disadvantage of SMAPE is its interpretability. While MAPE has a range of values from 0 to 100%, SMAPE has a range of values from 0 to 200%, with 0% being the best value.

A basic linear regression model [49] serves as a benchmark for better assessing the performance of the Random Forest Regressor and XGBoost Regressor. The results of this model are shown in the following Table 4. This table contains valuable metrics such as $R^{2}$, MSE, RMSE, MAE, and SMAPE, which reflect the performance of the regressor for each sensor. With these results, we can assess the strength of the relationship between the sensors and the output, as well as the accuracy of the model.

The CO_2_ sensor has the highest $R^{2}$ value of 0.60, indicating that it explains about 60% of the variability in the data. The NIR sensor also has a high $R^{2}$ value of 0.86, indicating a strong linear relationship between the sensor and the output. On the other hand, the pH sensor has the lowest $R^{2}$ value of 0.26, indicating a weaker linear relationship. The MSE values range from 5.50 for the CO_2_ sensor to 11.02 for the pH sensor, with the NIR sensor having the lowest value of 1.89. Similarly, the RMSE values range from 2.34 for the CO_2_ sensor to 3.32 for the pH sensor, with the NIR sensor having the lowest value of 1.37. The MAE values range from 1.70 for the CO_2_ sensor to 2.82 for the pH sensor, with the NIR sensor having the lowest value of 1.04. Finally, the SMAPE values range from 72.32% for the CO_2_ sensor to 121.95% for the pH sensor, with the NIR sensor having the lowest value of 63.27%. In summary, the results of the benchmark linear regression model show that the NIR sensor has the best performance in terms of $R^{2}$, MSE, RMSE, MAE, and SMAPE among the sensors used, while the pH sensor has the worst performance.

The results of the evaluation of the Random Forest Regressor are presented in Table 5.

The VOC sensor has the highest $R^{2}$ value of 0.99, indicating that it explains almost 99% of the variability in the data. The CO_2_ sensor also has a high $R^{2}$ value of 0.98, indicating a strong relationship between the sensor and the output. On the other hand, the Ethanol sensor has the lowest $R^{2}$ value of 0.56, indicating a weaker relationship. The MSE values range from 0.14 for the VOC sensor to 5.52 for the Ethanol sensor, with the CO_2_ sensor having the second lowest value of 0.34. Similarly, the RMSE values range from 0.37 for the VOC sensor to 2.35 for the Ethanol sensor, with the CO_2_ sensor having the second lowest value of 0.58. The MAE values range from 0.15 for the VOC sensor to 1.78 for the Ethanol sensor, with the CO_2_ sensor having the second lowest value of 0.23. Finally, the SMAPE values range from 12.52% for the VOC sensor to 93.38% for the Ethanol sensor, with the CO_2_ sensor having the second lowest value of 15.76%. In summary, The results of the Random Forest Regressor model show that the VOC sensor has the best performance in terms of $R^{2}$, MSE, RMSE, MAE, and SMAPE among the sensors used, while the Ethanol sensor has the worst performance.

The results of the XGBoost Regressor evaluation are shown in Table 6.

The VOC sensor has the highest $R^{2}$ value of 0.99, indicating that it explains almost 99% of the variability in the data. The CO_2_ sensor also has a high $R^{2}$ value of 0.97, indicating a strong relationship between the sensor and the output. On the other hand, the Ethanol sensor has the lowest $R^{2}$ value of 0.56, indicating a weaker relationship. The MSE values range from 0.14 for the VOC sensor to 5.51 for the Ethanol sensor, with the CO_2_ sensor having the second lowest value of 0.42. Similarly, the RMSE values range from 0.38 for the VOC sensor to 2.35 for the Ethanol sensor, with the CO_2_ sensor having the second lowest value of 0.65. The MAE values range from 0.18 for the VOC sensor to 1.72 for the Ethanol sensor, with the CO_2_ sensor having the second lowest value of 0.30. Finally, the SMAPE values range from 24.73% for the VOC sensor to 86.91% for the Ethanol sensor, with the CO_2_ sensor having the second lowest value of 30.99%. In summary, The results of the XGBoost Regressor model show that the VOC sensor has the best performance in terms of $R^{2}$, MSE, RMSE, and SMAPE among the sensors used. On the other hand, the Ethanol sensor has the worst performance in $R^{2}$ and SMAPE, while the NIR sensor has the worst performance in terms of MAE.

The NIR sensor has the best performance among the sensors used in the benchmark linear regression model, while the VOC sensor has the best performance in the Random Forest Regressor model and XGBoost Regressor model. The Ethanol sensor has the worst performance in all three models. Overall, the VOC sensor has the highest $R^{2}$ value and the best performance in terms of MSE, RMSE, MAE, and SMAPE among the sensors used in all three models.

## 5. Discussion

A fresh pizza was used to investigate whether low-cost sensors and machine learning methods are suitable for providing better information about the spoilage date. For this purpose, the sensors CO_2_, VOC, ethanol, pH and NIR were used in a defined measurement setup for storage tests. The data of the sensors served as an input for different regression models (linear regression, random forest regression and XGBoost regression). The evaluation revealed that it is possible to make accurate predictions about the fresh pizza’s spoilage date based on the low cost sensor data and a regression model. In particular, the sensors for VOC, CO_2_ and NIR proved to be particularly insightful. In combination with a random forest regressor or XGBoost regressor, they showed good results. Traditionally, determining the minimum shelf life date in the food industry involves empirical values and sample storage tests with microbiological and sensory analyses, with each company having its own approach. However, the integration of low-cost sensors and regression models represents a significant step towards a more sustainable and efficient food industry. This approach not only improves accuracy and cost-effectiveness but also supports the preservation of resources and the promotion of sustainable food consumption.

### 5.1. Findings

Regarding the question: *What are examples of the use of machine learning in the food industry regarding spoilage detection and what are their limitations?* Machine learning is being utilized in the food industry to improve spoilage detection and prevention in ready-to-eat pizza and other food products. Studies have analyzed the impact of packaging materials, modified atmospheres, and storage conditions on the sensory, chemical, and microbiological properties of pizza and its components like mozzarella cheese and vegetables. However, laboratory tests for these studies can be costly and time-consuming. Machine learning techniques such as neural networks, support vector machines, k-nearest neighbor algorithm, and image processing are being applied to address these limitations. These methods are used to classify food based on color and texture features, detect defects or stains, and determine the maturity and quality of fruits and vegetables. However, there are some challenges with these methods such as the cost of technology, the requirement for large data sets to train the models, and lower accuracy compared to traditional methods in some cases. Further research is needed to evaluate the efficacy of these methods in real-world scenarios.

Regarding the question: *What types of sensors provide the most relevant and accurate data for determining the shelf life of fresh pizza?* The evaluation conducted on the data of the five sensors (CO_2_, VOC, ethanol, pH, and NIR) indicate that VOC is the most relevant and accurate for determining the shelf life of fresh pizza. VOC performed best in two of the three regression models tested (Random Forest and XGBoost), and the CO_2_ sensor data was a close runner-up with similar results. NIR performed well in the linear regression model, but showed slight inferiority in the other two models. On the other hand, the data from ethanol and pH sensors had the poorest results and are considered unsuitable for predicting spoilage date. Hence, it is advised to use VOC, CO_2_, and NIR sensors instead for collecting useful data for determining the shelf life of fresh pizza.

Regarding the question: *What machine learning algorithm can be effectively utilized to predict the shelf life of fresh pizza using sensor data?* The evaluation of linear regression, random forest regression, and XGBoost regression models found that regression models are suitable for predicting spoilage and shelf life of fresh pizza. The best result was achieved with the Random Forest Regression using VOC sensor data, with an $R^{2}$ value of 0.99 and a SMAPE value of 12.52%. The second best was with the Random Forest Regression and CO_2_ sensor data, with an $R^{2}$ value of 0.98 and a SMAPE value of 15.76%. The third best was with the XGBoost Regression using VOC sensor data, with an $R^{2}$ value of 0.99 and a SMAPE value of 24.73%. The Random Forest Regressor performed slightly better than the XGBoost Regressor. But both are capable of accurately predicting the remaining shelf life of fresh pizza. This is evident from their superior performance compared to the benchmark linear regression, which showed significantly worse results across all sensor data.

### 5.2. Strengths and Weaknesses

The use of low-cost sensors, such as VOC or CO_2_, together with a regression model, such as Random Forest Regressor or XGBoost Regressor, provides a highly accurate and cost-effective way to determine the freshness of pizza. This allows for more precise predictions of the day of spoilage and the best-before date, ultimately reducing food waste and promoting sustainable food consumption. Food waste reduction is becoming increasingly important due to growing concerns over sustainability and the depletion of resources. By applying our approach, we can save valuable resources and have a positive impact on the environment.

Our approach to determine the freshness of pizza using low-cost sensors and regression models is limited in some aspects that need to be considered. Firstly, the training data used to develop the model were collected under controlled conditions, but real-world conditions such as temperature and humidity fluctuations can significantly impact the freshness of the pizza. Predictions based on the model are only accurate if the food is stored under similar conditions as the training data. This limitation restricts the general applicability of the model, particularly when applied to other stages of the food’s life cycle such as transportation or consumption by end-users. It would be crucial to collect additional information during these stages and add it to the model as extra features in order to address this issue. Additionally, fresh pizza is a complex food item made up of numerous ingredients, including various toppings, which may affect the accuracy of the model performance. To determine the approach’s robustness and confirm its validity, it is critical to evaluate it for other variants of pizza. We can increase the accuracy and effectiveness of the approach for determining the freshness of pizza by taking into account these limitations and making the necessary improvements.

### 5.3. Outlook

To achieve practical applicability of the integration of sensor data from VOC, CO_2_, and NIR, further research is required to address the limitations of the current proof-of-concept results. In order to improve data quality and enhance the accuracy of the prediction model, a comprehensive data collection and analysis approach should be adopted. This would involve expanding the model and data collection to encompass all phases of the pizza life cycle, including production, distribution, sales, and consumption. Additionally, life cycle phase should be integrated as an additional feature in the model to capture the variability in the gas sensor and NIR signals across the different phases. Further investigation on the predictive ability of the model on pizzas with various toppings would also enable the robustness and informative value of the sensor and NIR data to be evaluated. These efforts will facilitate the practical implementation of the integrated sensor data approach in everyday settings.

## Figures and Tables

**Table 1:** Example Subset VOC data.

Humidity	Temperature	VOC	Label
75.33	6.77	26081	7
75.30	6.77	26088	7
75.33	6.79	26091	7
75.32	6.77	26089	7
75.32	6.78	26086	7

**Table 2:** Best Model Parameters Random Forest Regressor.

Sensors	n_Estimators	Min_Samples_Split	Max_Depth
${\mathrm{CO}}_{2}$	20	2	None
**VOC**	10	10	None
**Ethanol**	50	2	5
**pH**	500	20	10
**NIR**	100	2	10

**Table 3:** Best Model Parameters XGBoost Regressor.

Sensors	n_Estimators	Max_Depth	Learning_Rate	Gamma
${\mathrm{CO}}_{2}$	20	10	1	10
**VOC**	20	10	0.5	0
**Ethanol**	1000	5	0.1	10
**pH**	50	5	0.1	10
**NIR**	100	5	0.1	0

**Table 4:** Performance Metrics Benchmark Linear Regression.

Sensors	$R^{2}$	MSE	RMSE	MAE	SMAPE
${\mathrm{CO}}_{2}$	0.60	5.50	2.34	1.70	72.32%
**VOC**	0.43	7.15	2.67	2.25	98.53%
**Ethanol**	0.40	7.49	2.74	2.26	114.95%
**pH**	0.26	11.02	3.32	2.82	121.95%
**NIR**	0.86	1.89	1.37	1.04	63.27%

**Table 5:** Performance Metrics Random Forest Regressor.

Sensors	$R^{2}$	MSE	RMSE	MAE	SMAPE
${\mathrm{CO}}_{2}$	0.98	0.34	0.58	0.23	15.76%
**VOC**	0.99	0.14	0.37	0.15	12.52%
**Ethanol**	0.56	5.52	2.35	1.78	93.38%
**pH**	0.76	3.65	1.91	1.18	59.92%
**NIR**	0.97	0.43	0.66	0.32	30.80%

**Table 6:** Performance Metrics XGBoost Regressor.

Sensors	$R^{2}$	MSE	RMSE	MAE	SMAPE
${\mathrm{CO}}_{2}$	0.97	0.42	0.65	0.30	30.99%
**VOC**	0.99	0.14	0.38	0.18	24.73%
**Ethanol**	0.56	5.51	2.35	1.72	86.91%
**pH**	0.76	3.56	1.89	1.22	64.30%
**NIR**	0.97	0.48	0.69	0.40	35.13%

## Data Availability

Data available on request.
